# Effect of thalidomide on the expression of vascular endothelial growth factor in a rat model of liver regeneration

**DOI:** 10.3892/ol.2012.1089

**Published:** 2012-12-20

**Authors:** KUO-CHEN HUNG, PEI-MIN HSIEH, KUN-LIN YANG, KAI-JEN LIN, YAW-SEN CHEN, CHIH-HSIN HUNG

**Affiliations:** 1 Institute of Biotechnology and Chemical Engineering, I-Shou University;; 2 Department of Surgery, Division of General Surgery, Yuan’s General Hospital;; 3 Department of Surgery, Division of General Surgery, E-DA Hospital/I-Shou University, Kaohsiung;; 4 Institute of Basic Medical Sciences College of Medicine, National Cheng Kung University, Tainan;; 5 Department of Pathology, E-DA Hospital/I-Shou University, Kaohsiung, Taiwan, R.O.C.

**Keywords:** angiogenesis, hepatectomy, liver regeneration, thalidomide, vascular endothelial growth factor

## Abstract

Liver regeneration is an angiogenesis-associated phenomenon. The present study investigated the influence of thalidomide, an antiangiogenic agent, on vascular endothelial growth factor (VEGF) expression and liver regeneration after 70% partial hepatectomy (PH) in rats. PH was performed on 50 rats dosed with either thalidomide (100 mg/kg) or a vehicle (controls) by intragastric administration. Serial changes in hepatic microcirculation were evaluated by laser Doppler flowmetry. The VEGF expression in liver tissue was assessed by immunohistochemical study and western blot analysis. Following hepatectomy, the liver regeneration rate increased markedly and reached a peak at 96 h in the two groups. Thalidomide did not affect the overall restoration of liver mass, although a delay in cell proliferation was observed. Prior to PH, the liver microcirculation in rats treated with thalidomide for 2 days was comparatively less than that in their corresponding controls; however, no significant difference between the groups was detected at any time-point following PH. Western blotting showed that the expression of VEGF was upregulated by hepatectomy and the expression levels in the two groups were equal at all studied time-points. The immunohistochemical staining revealed a waved pattern of VEGF expression which advanced from the periportal to pericentral area in both groups, but a slower advancement was detected in thalidomide-treated rats. In conclusion, thalidomide exerted no significant effects on the expression of VEGF and did not impair the overall restoration of liver mass in a rat model of PH-induced liver regeneration, providing supportive evidence for its use as an adjunct treatment modality for liver cancers.

## Introduction

Liver regeneration is a tissue repair response of the liver following damage due to various causes, including viral infection, chemical intoxication and partial hepatectomy (PH). Although the exact underlying mechanisms have not been fully characterized, the process is acknowledged to be tightly regulated through controlled delivery of ‘start and stop’ signals, including numerous cytokines and growth factors, to maintain a constant liver-to-body mass ratio (1–3). A number of the growth factors involved in a regenerating liver are known for their angiogenic properties (4). Among the various angiogenic factors that have been identified, including basic fibroblast growth factor (bFGF), vascular endothelial growth factor (VEGF) and platelet-derived growth factor (PDGF), VEGF has been demonstrated to be a major angiogenic factor following PH (5,6).

Thalidomide, α-*N*-phthalimido-glutarimide, was initially marketed as a sedative and antinausea medicine in the 1950s, but was withdrawn due to teratogenicity (7). Unexpectedly, it has become the subject of intensive investigation in oncology since its antiangiogenic properties were first demonstrated in 1994 (8). In that study, the bFGF-induced neovascularization in rabbit corneas was significantly reduced by thalidomide. This drug has also been shown to inhibit VEGF-induced angiogenesis (9,10). In addition to its antiangiogenic effect, an immunomodulatory function is also a potential mechanism of the anticancer activity of thalidomide. To date, the effectiveness of thalidomide for treating neoplastic disorders has been confirmed in diseases such as multiple myeloma (11) and Kaposi’s sarcoma (12). In addition, thalidomide has been tentatively used for the treatment of advanced hepatocellular carcinoma (13–16).

Antiangiogenic factors have been demonstrated to reduce the formation of new blood vessels (17), resulting in slower tumor growth or even tumor regression. Therefore, the combination of antiangiogenic strategies with liver resection is a promising approach to treat primary and metastatic liver cancers, such as hepatocellular carcinoma and colorectal cancer. Post-hepatectomy liver failure develops if liver regeneration is impaired, especially in antiangiogenic condition. However, the effect of the antiangiogenic agent on liver regeneration has not been fully clarified. In the present study, we investigated the effect of thalidomide on VEGF expression and liver regeneration in rats following 70% PH.

## Materials and methods

### Animals

Male Sprague-Dawley rats initially weighing 250–300 g were used. All animals were housed in a temperature and humidity controlled environment, and they received humane care with free access to standard chow and water throughout the study period. The protocols in this study were submitted to and approved by the E-Da Hospital (Taiwan) Institutional Animal Care and Use Committee (IACUC-97007). All animal procedures were in compliance with our institutional guidelines.

### Experimental design

A total of 50 rats were subjected to 70% PH and equally divided into two groups: the control and thalidomide groups. Two days prior to PH, rats in the thalidomide group were daily administered thalidomide (100 mg/kg, TTY BioPharm, Taipei, Taiwan) in olive oil by intragastric administration. Control rats received olive oil only. Animals in the two groups were equally divided into 5 subgroups according to observation intervals, which were 0, 48, 96, 144 and 192 h after PH.

### PH

Liver regeneration was induced by 70% PH as described by Higgins and Anderson (18). Animals were anesthetized with ketamine (100 mg/kg, intraperitoneal injection). After a midline laparotomy, the liver was exposed and the left and medial lobes were ligated (4-0 silk) and resected. Glucose solution (5 ml; 5%; 37°C) was injected into the abdominal cavity and the abdominal wound was closed in two layers with 4-0 silk. The resected liver was termed ‘quiescent liver’ in this study.

### Hepatic regeneration rate

The rate of liver regeneration was evaluated using the formula of Kwon *et al*(19): Hepatic regeneration rate (%) = D/E × 100, where D is the weight of the liver per 100 g of body weight at death and E is the estimated liver weight per 100 g body weight prior to hepatectomy, which was calculated from the weight of resected liver (R); E = R/0.7.

### Laser Doppler flowmetry analysis of microcirculation

The principle of laser Doppler flowmetry combines laser technology with the Doppler effect caused by the movement of red blood cells in the microcirculation to estimate red blood cell flux (20). The strength of this technique is in observing changes in flow, either over time or over an area of the exposed tissue. Before 70% PH and animal sacrifice, the surface of the liver was scanned by a Moor LDI 2 imager (Moor Instruments Ltd., Devon, UK) to assess the perfusion hemodynamics. The Doppler shift is proportional to a blood flow-related variable and is expressed in arbitrary perfusion units (PU). Microcirculation density was quantified using software provided by the manufacturer (Moor LDI system software V5).

### Western blot analysis

Livers were homogenized by Ultrasonic cell disruptor (Microson™ XL-2000; Misonix, Farmingdale, NY, USA) in tissue protein extraction buffer (T-PER^®^, Pierce, Rockford, IL, USA) containing protease inhibitors (Protease Inhibitor Cocktail 100X, Pierce) and the homogenate was centrifuged to obtain the supernatant. Protein concentrations were determined and the samples were subjected to sodium dodecyl sulfate/polyacrylamide gel electrophoresis and transferred to a nitrocellulose membrane (ECL, Amersham, Buckinghamshire, UK). After blocking and washing, blots were incubated overnight at 4°C with rabbit affinity-purified antibodies against proliferating cell nuclear antigen (PCNA; dilution, 1:1,000; Epitomics, Burlingame, CA, USA), VEGF (dilution, 1:1,000; Santa Cruz Biotechnology, Inc., Santa Cruz, CA, USA) or β-actin (dilution, 1:100; Sigma, St. Louis, MO, USA). The blots were washed and incubated with horseradish peroxidase (HRP)-conjugated secondary antibodies for 1 h. Finally, the signals were detected using an enhanced chemiluminescence detection kit (Amersham, Piscataway, NJ, USA). The chemiluminescent signal was captured by a UVP BioSpectrum500 imaging system (UVP, Upland, CA, USA).

### Immunohistochemistry

Fresh liver samples were immediately immersed into 10% neutral formalin. Paraffin-embedded liver samples were cut into 5-*μ*m sections. Liver sections were de-paraffinized, rehydrated and placed in citrate buffer (10 mM, pH 6.0) and microwaved twice for 7 min to improve staining by antigen unmasking. After dewaxing and rehydration, liver sections were placed in citrate buffer (10 mM, pH 6.0) and microwaved twice for 7 min to improve staining by antigen unmasking. The activity of endogenous peroxidase was removed by incubation with 3% H_2_O_2_ for 15 min at room temperature. VEGF was identified by rabbit anti-rat VEGF polyclonal antibody (dilution, 1:500; SC-152, Santa Cruz Biotechnology, Inc.) followed by HRP-conjugated goat anti-rabbit secondary antibody (Dako™ REAL™ EnVision Detection System, K5007; Carpinteria, CA, USA). Positive signals were shown by 3,3′-diaminobenzidine (DAB) response. Sections were then counterstained with hematoxylin.

### Statistical analysis

Student’s t-test was used to compare sample means with paired or unpaired controls, as appropriate. Results are expressed as means ± SEM. P<0.05 was considered to indicate a statistically significant result.

## Results

### Liver regeneration rate

**Figure 1:**
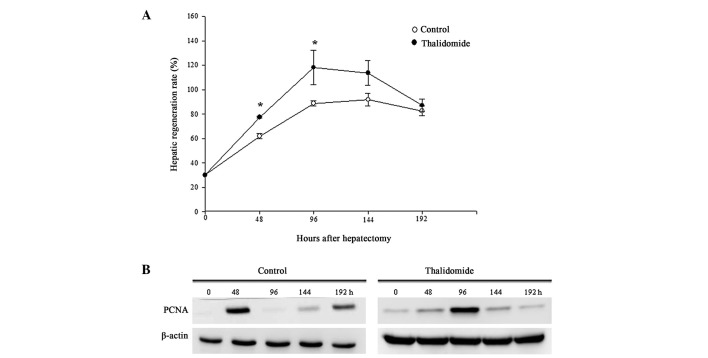
Liver regeneration following partial hepatectomy. (A) Hepatic regeneration rate (%) = D/E × 100, where D is the weight of the liver per 100 g of body weight when sacrificed and E represents the estimated liver weight per 100 g body weight prior to hepatectomy, which was calculated from resected liver weight (R); E = R/0.7. Results shown are the means ± SEM of data from 5 different rats/group per time-point. ^*^P<0.05 versus 0 h control. (B) Densitometric analysis of hepatic PCNA protein expression. The expression of β-actin was used as loading control. PCNA, proliferating cell nuclear antigen.

All the experimental rats survived the 70% liver resection and thalidomide treatment. Following hepatectomy, the restituted liver mass in the two groups markedly increased with a peak at 96 h and then declined (Fig. 1A). Unexpectedly, the calculated regeneration rate in the thalidomide group at the first two post-hepatectomy time-points (48 and 96 h) was significantly higher than that in the control rats, while no difference between the groups was found at the subsequent time-points.

### Expression of PCNA

PCNA is a protein marker for DNA synthesis and is commonly used as an indicator for cell proliferation. At resting state, weak expression of PCNA was detected in both groups. Following hepatectomy, the expression level of PCNA increased significantly and reached a peak at 48 h in the control group, 96 h in the thalidomide group and declined abruptly thereafter (Fig. 1B).

### Hepatic microcirculation

**Figure 2:**
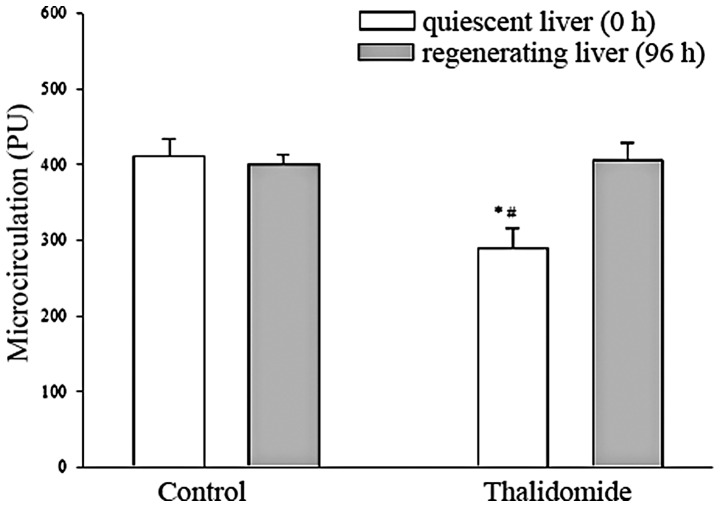
Laser Doppler flowmetry of the hepatic microcirculation. Hepatic blood flow was measured before hepatectomy (0 h, quiescent liver) and at 96 h post-PH (regenerating liver). The liver microcirculation at 0 h in the thalidomide group was obtained from rats treated with thalidomide for 48 h before measurement. ^*^P<0.05 versus quiescent liver in control group; ^#^P<0.05 versus thalidomide-treated regenerating liver at 96 h. PH, partial hepatectomy.

To quantify the circulatory effect of thalidomide on liver regeneration, hepatic blood flow was assessed by laser Doppler flowmetry before PH (0 h, quiescent liver) and sacrifice (regenerating liver). Prior to PH, the hepatic microcirculation in rats treated with thalidomide for 2 days was comparatively less than that in their corresponding controls (Fig. 2). However, no significant difference in blood flow in the remnant liver between the control and thalidomide groups was detected at any studied time-point.

### Western blot analysis of VEGF

**Figure 3:**
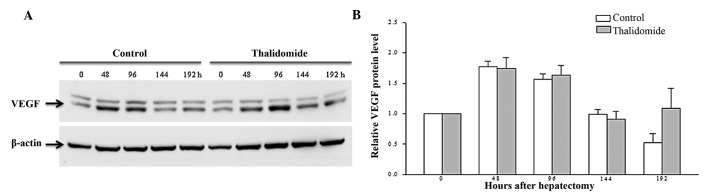
Expression of VEGF in rat liver at different time-points following partial hepatectomy. The expression of VEGF was normalized against that of β-actin. (A) Representative immunoblot. (B) Densitometric analysis. Results shown are the mean ± SEM of data from 5 different rats/group per time-point. ^*^P<0.05 versus 0 h control. The expression at 0 h is arbitrarily normalized to 1. VEGF, vascular endothelial growth factor.

Prior to liver resection (0 h), a low expression level of VEGF was detected in control rats and rats treated with thalidomide for 48 h (Fig. 3). Hepatectomy induced marked expression of VEGF, which peaked at 48–96 h and declined rapidly in the two groups. No significant difference in the expression level between the groups at any studied time-point was found.

### Immunohistochemical staining of VEGF

**Figure 4:**
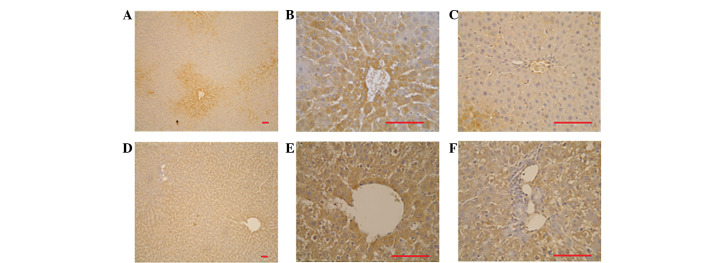
Immunohistochemistry for VEGF in regenerating liver at 96 h after partial hepatectomy. VEGF was stained with rabbit anti-rat VEGF antibody followed by HRP-conjugated goat anti-rabbit secondary antibody. Positive signals were shown by DAB response. Sections were then counterstained with hematoxylin. Control group (A) pericentral area (×40); (B) pericentral area (×400); (C) periportal area (×400). Thalidomide group (D) pericentral and periportal areas (×40); (E) pericentral area (×400); (F) periportal area (×400). Scale bars, 50 *μ*m. VEGF, vascular endothelial growth factor; HRP, horseradish peroxidase; DAB, 3,3′-diaminobenzidine.

Positive VEGF immunoreactivity was mainly localized in the cytoplasm of hepatocytes. Prior to PH, faint staining was observed in the two groups. At 48 h after PH, VEGF was mainly expressed in the periportal area in both groups (data not shown). At 96 h, the positive immunoreactivity was limited to the pericentral area in the control group (Fig. 4A–C), while observed in peri-central and periportal hepatocytes in the thalidomide group (Fig. 4D–F). At the subsequent time-points, markedly weaker expression of VEGF was observed and mostly located in the pericentral area in the two groups (data not shown).

## Discussion

The present study demonstrated that thalidomide delayed the PH-induced hepatic cell proliferation but did not impair the overall liver regeneration. In addition, the PH-induced upregulation of VEGF was not inhibited by thalidomide.

The maximal expression of PCNA, a marker for DNA synthesis, was observed to occur at 48 h post-hepatectomy in the control group of this study. However, the peak for hepatocyte proliferation during liver regeneration in the rat as determined by Ki-67 or 5-bromodeoxyuridine (5-BrdU) labeling is at 24 h (1). There are at least two possible explanations for this discrepancy. One is that 24 h was not one of the selected time-points in our study. The other is the methodologies used in different studies. The approach we employed in this study was detection of the overall PCNA expression in liver homogenate, which is derived from various types of cells with different proliferation rates. By contrast, Ki-67 staining is used to determine the growth of a specific cell population, such as hepatocytes in the liver. In the present study, the maximal PCNA expression detected in the thalidomide group was 96 h after liver resection, a significant delay as compared with control rats; nevertheless, the overall restoration of liver mass was not affected.

The significance for the observed transient greater liver regeneration rate in the thalidomide group requires further investigation. However, we speculated that it may be due to the non-specific effect of thalidomide, such as increasing the water content in liver tissue based on the transient watery appearance of thalidomide-treated liver (our unpublished observation). If so, the liver regeneration rate may thus be overestimated.

Laser Doppler flowmetry is a technique for the non-invasive blood flow monitoring and is considered to be a suitable technique for the analysis of hepatic microcirculation (20–22). In the present study, thalidomide impaired hepatic micro-circulation in the quiescent, but not regenerating, liver. The reduced blood flow in the thalidomide-treated quiescent liver may be associated with the inhibitory effect of thalidomide on the release of tumor necrosis factor (TNF)-α and nitric oxide, two potent vasodilators, as suggested by a previous study in which thalidomide ameliorated the portal pressure and hyper-dynamic circulation in partially portal vein-ligated rats by reducing TNF-α and nitric oxide production (23). After PH, this inhibitory effect of thalidomide was eliminated by rapid release of TNF-α (1), resulting in similar hepatic microcirculation in the two groups.

VEGF is an important factor in the early phase of liver regeneration (24). In this study, treatment with thalidomide for 48 h before PH did not affect the expression of VEGF, as evidenced by the insignificant difference in the expression level between control and thalidomide groups at 0 h. Following PH, VEGF was markedly upregulated and the expression profile during the regenerative process was similar in the two groups. These observations suggest that thalidomide exerts no significant effect on the expression of VEGF either in quiescent liver or in PH-induced regenerating liver. The immunohistochemical result showing that VEGF was predominantly expressed in the periportal hepatocytes at 48 h post-PH is consistent with a previous study (5). At 96 h, the positive staining was observed only in the pericentral area in the control group, while observed in the periportal and pericentral areas in the thalidomide group. This time-dependent alteration in the main expression site in the liver suggests a waved pattern for the expression of VEGF, which advances from the periportal to pericentral area. Although the significance for the observed difference in the expression areas at 96 h between groups requires further studies for clarification, we hypothesize that that it may reflect the slower angiogenesis in thalidomide-treated rats.

In conclusion, our results demonstrate that thalidomide exerts no significant effect on the expression of VEGF and does not impair the overall PH-induced restoration of liver mass, providing supportive evidence that thalidomide may be used as an adjunct treatment modality for liver cancers.
